# Supplementary material for: EnzML: multi-label prediction of enzyme classes using InterPro signatures
Source: BMC Bioinformatics. 2012 Apr 25;13:61. doi: 10.1186/1471-2105-13-61 (PMC3483700; doi:10.1186/1471-2105-13-61)
Supplement: Addtional file 5 — The Java code to format the data files, evaluate and predict. The file enzml_java_code.tar.gz contains the Java code used to format database data to ARFF and XML formats, to execute cross and train-test (jackknife) evaluations and to record evaluation results to database. More information is included in the readme.txt file and the Javadoc files. The code can be used with a MySQL database. To use a different database software, other JDBC drivers might be required. [file 1471-2105-13-61-S5.gz › java_code/enzml2011/doc/test/mulan/attributesfilter/AttributesFilteredArffTest.html]

AttributesFilteredArffTest


---


|  |  |  |
| --- | --- | --- |
| **Overview** \| **Package** \| **Class** \| **Use** \| **Tree** \| **Deprecated** \| **Index** \| **Help** | |  |
| **PREV CLASS**   **NEXT CLASS** | **FRAMES**    **NO FRAMES**     **All Classes** |  |
| SUMMARY: NESTED \| FIELD \| CONSTR \| METHOD | DETAIL: FIELD \| CONSTR \| METHOD |  |


---


## test.mulan.attributesfilter Class AttributesFilteredArffTest

```
java.lang.Object
  junit.framework.Assert
      junit.framework.TestCase
          test.mulan.attributesfilter.AttributesFilteredArffTest
```

**All Implemented Interfaces:**: junit.framework.Test

---

``` public class AttributesFilteredArffTest extends junit.framework.TestCase ```

---

| **Field Summary** | |
| --- | --- |
| `static int` | `TEST_ROLE` |
| `static int` | `TRAIN_ROLE` |


**Constructor Summary**

`AttributesFilteredArffTest()`


| **Method Summary** | |
| --- | --- |
| `static java.lang.String` | `getCorrectAttributes()` |
| `static java.lang.String` | `getCorrectClasses()` |
| `static int` | `getFileRole(java.lang.String fileName)`             Returns the role ("train" or "test") of the file for a potential train/test evaluation. |
| `static int[]` | `getNewFilteredArffs()`             Get the new, filtered, train and test arff files |
| `static void` | `testANewFilteredFileContent(int role, int newId, java.lang.String[] content)` |
| `void` | `testArffName()` |
| `void` | `testNewTestsetFile()` |
| `void` | `testNewTrainsetFile()` |

**Methods inherited from class junit.framework.TestCase**

`countTestCases, getName, run, run, runBare, setName, toString`

**Methods inherited from class junit.framework.Assert**

`assertEquals, assertEquals, assertEquals, assertEquals, assertEquals, assertEquals, assertEquals, assertEquals, assertEquals, assertEquals, assertEquals, assertEquals, assertEquals, assertEquals, assertEquals, assertEquals, assertEquals, assertEquals, assertEquals, assertEquals, assertFalse, assertFalse, assertNotNull, assertNotNull, assertNotSame, assertNotSame, assertNull, assertNull, assertSame, assertSame, assertTrue, assertTrue, fail, fail, failNotEquals, failNotSame, failSame, format`

**Methods inherited from class java.lang.Object**

`equals, getClass, hashCode, notify, notifyAll, wait, wait, wait`

**Field Detail**

### TRAIN\_ROLE

```
public static final int TRAIN_ROLE
```

**See Also:**: Constant Field Values

---


### TEST\_ROLE

```
public static final int TEST_ROLE
```

**See Also:**: Constant Field Values


**Constructor Detail**

### AttributesFilteredArffTest

```
public AttributesFilteredArffTest()
```


**Method Detail**

### testArffName

```
public void testArffName()
```

---


### testNewTestsetFile

```
public void testNewTestsetFile()
```

---


### testNewTrainsetFile

```
public void testNewTrainsetFile()
```

---


### getCorrectAttributes

```
public static java.lang.String getCorrectAttributes()
```

---


### getCorrectClasses

```
public static java.lang.String getCorrectClasses()
```

---


### getFileRole

```
public static int getFileRole(java.lang.String fileName)
```

:   Returns the role ("train" or "test") of the file for a potential
    train/test evaluation. A filtered train file has the same id for the
    filtered file \*and\* for the attributes. A filtered test file has
    different id for the filtered file and the attributes (because the
    attributes come from the old train file).

    :   **Parameters:**: `fileName` - a filtered arff file name **Returns:**: 0 if a train file, 1 if a test file

---


### getNewFilteredArffs

```
public static int[] getNewFilteredArffs()
```

:   Get the new, filtered, train and test arff files

    :   **Returns:**: an array where the first element is the new train arff and the second element is the new test arff

---


### testANewFilteredFileContent

```
public static void testANewFilteredFileContent(int role,
                                               int newId,
                                               java.lang.String[] content)
```


---


|  |  |  |
| --- | --- | --- |
| **Overview** \| **Package** \| **Class** \| **Use** \| **Tree** \| **Deprecated** \| **Index** \| **Help** | |  |
| **PREV CLASS**   **NEXT CLASS** | **FRAMES**    **NO FRAMES**     **All Classes** |  |
| SUMMARY: NESTED \| FIELD \| CONSTR \| METHOD | DETAIL: FIELD \| CONSTR \| METHOD |  |


---
